# Single photon event-driven 3D imaging

**DOI:** 10.1038/s44172-025-00555-7

**Published:** 2025-11-29

**Authors:** Alex Vicente Sola, Matthias Aquilina, Paul Kirkland, Ashley Lyons

**Affiliations:** 1 Neuromorphic Sensor Signal Processing Lab, Department of Electronic and Electrical Engineering, University of Strathclyde, Glasgow, UK; 2 School of Physics and Astronomy, University of Glasgow, Glasgow, UK; 3 International Centre for Neuromorphic Systems, Western Sydney University, Penrith, Australia

**Keywords:** Electrical and electronic engineering, Single photons and quantum effects

## Abstract

Event-based imaging is at the forefront of high-speed sensing applications due to the low latency of asynchronous detection and low data volume. Conventionally, events used in this form of sensing correspond to changes in intensity on the microsecond scale, this inherently makes the approach incompatible with scenarios where single photon detection is required such as single photon Light Detection and Ranging (LiDAR), and low-light-level imaging. Here we propose a new imaging modality which is driven instead by events generated from the detection of individual photons. We use a form of single-pixel imaging in which information from a 3-Dimensional scene is encoded entirely in the time-of-arrival of the photons such that each detected photon can be used to update an estimation of all transverse positions simultaneously. The image reconstruction is performed by a Spiking Convolutional Neural Network (SCNN) which has a natural complementarity with single photon detection that allows the scheme to run fully asynchronously and be driven by the detection of each individual photon. Our approach for processing LiDAR information asynchronously, driven by each detected photon, has the potential for minimal latency 3D imaging and sensing even in weak light conditions with applications in high speed target detection and robotics.

## Abstract (web summary)

Alex Vicente Sola and authors introduce a new high-speed LiDAR-based imaging modality driven by the time-of-arrival of individually detected photons and reconstructed by a spiking convolutional neural network, allowing the system to run fully asynchronously.

## Introduction

Real-time 3D scene analysis is essential for advancements in autonomous vehicles^1,2^, robotics^3^, and other machine vision tasks^4^. However, traditional imaging methods often struggle with high-speed, high-frame-rate capture, particularly in low-light conditions or dynamic environments^5,6^. This struggle is typically due to a bottleneck at the processing stage caused by the large volume of discrete data being sensed over many pixels. Single-pixel imaging (SPI) provides considerable advantages in terms of data volume and also offers improvements in latency, spectral range, sensitivity, and timing resolution^7,8^. These benefits are currently enhancing applications ranging from biomedical imaging to LiDAR (Light Detection and Ranging).

In contrast with conventional imaging with a pixelated detector, SPI is not able to retrieve image information in a single measurement. This is often circumvented through sequential dynamic measurements, either by scanning the detector or with structured illumination/detection, typically using a Spatial Light Modulator. Although advantages can be found in the reduction of the number of required measurements through compressive sensing^9,10^, these approaches nevertheless require the full measurement routine to be completed before the image can be reconstructed fully. This restricts the effective frame rate of such SPI schemes and scales linearly with the number of pixels in the final image.

Influential studies by Turpin et al.^11^ and Kirkland et al.^12^ have shown the feasibility of using single-pixel detection combined with flood illumination for 3D imaging-overcoming the inverse retrieval problem with a data-driven approach-and the potential of Spiking Neural Networks (SNNs) for image processing, respectively. Instead of measuring spatial information directly, these studies encoded information of the full 3D environment into the time-of-flight of the photons such that it could be recovered from the measured temporal histograms. The same principle has been used since to increase the spatial resolution of low pixel count sensors for pose estimation^13,14^ and identify individuals without forming distinct images^15^. However, these methods heavily depend on fixed-rate histograms, thus failing to fully exploit the benefits of real, dynamic event-based sensing and processing. Such reliance on discrete histograms limits the potential for real-time applications and undermines the dynamic nature of event-based data.

Therefore, to fully exploit the benefits of the event-based nature of single photon detection, this work introduces the first end-to-end, event-based neuromorphic system for dynamic 3D depth-map reconstruction, named Single-Photon Event-Driven Imaging (SPEDI).

SPEDI tackles the complex task of 3D scene reconstruction using a commercial, off-the-shelf, event-driven Single Photon Avalanche Diode (SPAD) sensor. This sensor delivers a continuous stream of photon-event-driven 1D Time of Flight (ToF) data, enabling dynamic image capture. The reconstruction process utilises a Spiking Convolutional Neural Network (SCNN), which processes the event-driven data asynchronously and sparsely through binary spikes, inspired by the efficiency of biological neural circuits. This approach demonstrates how 3D scene reconstruction can be achieved from photon-event-driven 1D data in a fully event-driven, neuromorphic manner, showcasing the potential for more efficient and accurate imaging technologies.

SPEDI introduces a photon-event-driven 3D computational imaging approach that eliminates the need for structured light and histogram collection by directly processing photon arrival times. This methodology not only matches the reconstruction results of conventional data-driven neural network approaches using similar experimental schemes with fixed-rate histograms but also has the potential to reduce power consumption, computational load, and latency considerably. These potential reductions are primarily due to the elimination of histogramming and other traditional preprocessing steps, which are typically resource-intensive. Such improvements are particularly beneficial in dynamic environments where rapid image processing is critical. Furthermore, SPEDI benefits from a data acquisition scheme where each measured photon can be used to update the inferred 3D reconstruction as a whole. This is in contrast to conventional imaging with a pixelated detector array, where at least one photon per pixel would be required to update all transverse positions in the scene and the sequential measurements needed for typical single-pixel imaging approaches. This allows SPEDI to run fully asynchronously and be triggered by a single photon detection event.

Moreover, SPEDI’s development is especially timely, given the growing interest in Spiking Neural Networks (SNNs) for SPAD (Single-Photon Avalanche Diode) data processing^16–18^. This interest is spurred by the potential for enhanced efficiency and reduced latency. Recent studies have explored the application of these advancements in areas such as fluorescence lifetime imaging^19^ and have made efforts to integrate SNNs directly into SPAD sensors for timestamp compression^20^. Specifically, the later uses an SNN to infer the arrival time of a detected pulse rather than assigning an electronically measured timestamp to each photon thereby circumventing the need for an electronic Time-to-Digital Converter (TDC) for each pixel^20^. In contrast, SPEDI uses measured timestamps from a TDC, but trains the SNN directly and with real data, as opposed to ref. ^20^ which uses conversion methods on NengoDL and synthetic data. Later sections explain how converted networks process data synchronously, therefore, this work is the first to test the feasibility of a fully event-based processing pipeline. In conclusion, these advancements are complementary to the benefits of SPEDI, and suggest the potential for further enhancements in power, latency, and computational efficiency. Together, these factors position SPEDI at the forefront of next-generation imaging technologies, offering substantial benefits across various applications.

**Fig. 1:**
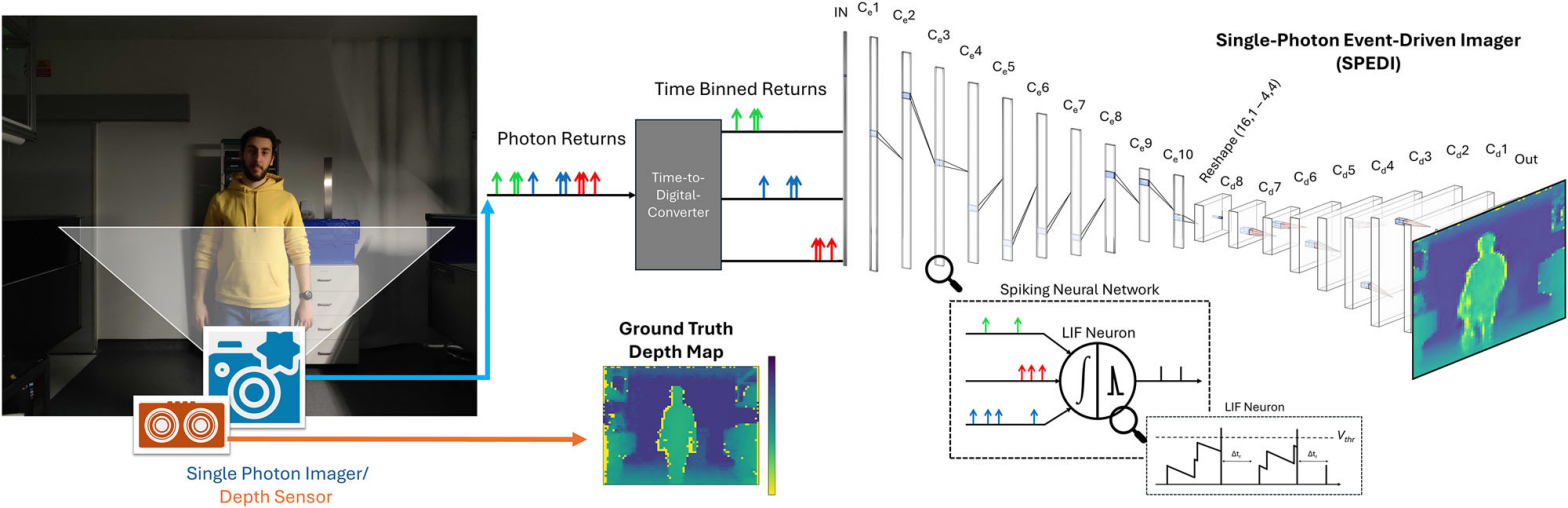
An illustration of the single-photon event-driven imaging system for dynamic 3D scene reconstruction. A single-photon imager captures light information, with each photon’s arrival time precisely recorded by a time-to-digital converter (TDC). Photon events of the same colour represent those which are collapsed into the same time-stamp by the TDC. These timestamped events trigger corresponding spikes in the relevant time bin, encoding information which feeds into a SCNN. The SCNN's leaky integrate-and-fire neurons accumulate these spikes over time, with internal thresholds driving information propagation. This fully event-based, spike-driven processing allows the system to dynamically reconstruct the 3D depth map of the scene with each incoming photon. 3D depth map ground truths for comparison are captured with the use of a depth sensor. The person in the image consented to the use of the photograph.

The SPEDI system, depicted in Fig. 1, exemplifies a cutting-edge, event-driven neuromorphic approach to 3D scene reconstruction. By directly processing photon returns through an SCNN and eliminating fixed-rate temporal accumulation, SPEDI leverages the dynamic processing capabilities of SNNs to overcome traditional SPI limitations and enhance imaging technology robustness. The results section will present how this novel approach allows for interpreting 1D data from single photons for accurate 3D scene reconstruction, challenging traditional expectations. Additionally, to validate these findings, a nearest neighbours approach serves as a baseline to ensure that the depth maps generated by SPEDI accurately reflect unseen test data, rather than simply reproducing memorised training patterns.

## Results

### Single-pixel event-based 3D imaging data collection

Building on traditional imaging methods, event-based single-pixel imaging represents a considerable leap forward. By merging the rapid capture capabilities of flood illumination with the detailed, photon-event-driven data from single-pixel detectors, this approach offers a comprehensive view of dynamic 3D scenes.

This study introduces SPEDI, an innovative approach employing a commercial-off-the-shelf (COTS) Flash LiDAR system equipped with the ‘SPAD23’ array from Pi Imaging^21,22^. The array features 23 pixels, each integrated with Time-Correlated Single Photon Counting (TCSPC) electronics that offer a 20-picosecond resolution for photon event timestamps. These are streamed continuously through a First In First Out buffer, enabling the unique asynchronous readout capability of the SPAD23. In this work, the outputs from all pixels are combined into a single asynchronous channel. This not only increases the overall detection efficiency of our system but also avoids effects arising from the dead time of individual SPAD detectors.

**Fig. 2:**
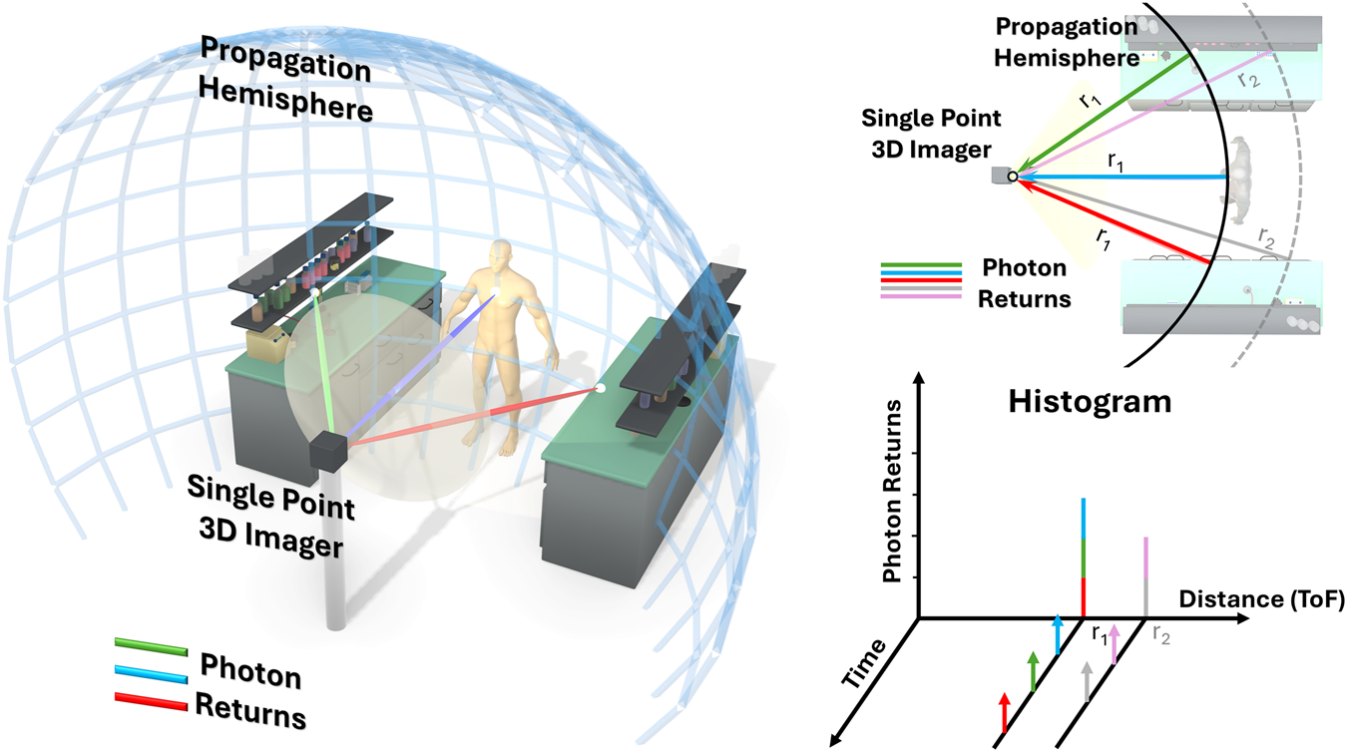
Visualisation of the SPI system during the data acquisition stage. This employs a flash illumination technique depicted as a transparent cone of light. Coloured lines represent the photon returns, demonstrating the multiplicity of return times that correspond to the same time-of-flight (ToF), such as points *r*_1_ and *r*_2_ on the propagation hemisphere. This illustrates the potential for each photon return to convey information about the entire 3D scene, a feature we exploit. The continuous event-based nature of the photon returns is captured, highlighting when they occur in time, and indicating the accumulation process that forms a ToF histogram.

The Flash LiDAR system utilises a  ~100 femtosecond pulsed laser with an 80 MHz repetition rate and a centre wavelength of 520 nm. A comprehensive dataset that includes various real-world complexities has been collected, which allows for a thorough evaluation of the SNN models. Ground-truth depth maps have been captured using an Intel RealSense 3D camera, which provides benchmarks against traditional image processing methods. Figure 2 illustrates the data collection process, underscoring both the challenge and advantage of SPI and the event-driven data collection employed by SPEDI, as well as the transformation of this data into Time of Flight histograms for discrete processing methods.

To further simulate realistic conditions and enhance the robustness of our evaluation, the dataset was enriched with a variety of background settings. This ensured that the learning algorithms could discern not only the histogram patterns of the foreground but also the intricate features of different background elements. It has previously been demonstrated that multiple photon scattering events incorporating static objects in the background contribute considerably to the overall image reconstruction quality^23^. To introduce substantial complexity to the task, three distinct background environments were incorporated, and the subject was recorded moving through the scene. This allowed us to observe how effectively the network could handle both movement within the scene and transitions between different backgrounds. A selection of snapshots from the data collection, along with their corresponding histograms, is presented to illustrate the complexity of the scenarios faced by the network. Given that the motion captured corresponds to natural human motion, speeds of 48 Hz or higher are considered slow motion and are arguably enough to avoid motion blur. The sensor acquires events at a frequency of 80 MHz, a time resolution which is orders of magnitude superior to typical slow motion sensors. When these events are accumulated in frames for non-spiking processing (CNN), it is done at an average frequency of 52 Hz, which is a standard slow motion frame rate. When processing fully event-based (SCNN), latency can go down to the original 80 MHz, but for simulation purposes in synchronous hardware (CPU/GPU) the average frequency used is 520 Hz.

### Event-driven spiking convolutional neural network

In this study, we present SPEDI, an SCNN architecture that combines the advantages of Convolutional Neural Networks (CNN) for feature extraction, the efficiency of encoder-decoder architectures, and the temporal adaptability of SNNs for asynchronous signal processing. An overview of the network layout was illustrated within Fig. 1, where the translation architecture of the network is highlighted. SPEDI is designed to accurately convert temporally resolved photon detections into depth maps. Using one-dimensional convolutions with spiking neurons, the architecture transforms photon timings into a structured representation similar to histograms. This encodes spatial and temporal variations that represent scene elements and occlusions, eliminating the need for discrete, fixed-rate histograms. An Encoder-Decoder configuration then maps these detailed encodings into a compact latent space, subsequently processed by a two-dimensional convolutional decoder that leverages spatial correlations in natural images.

Harnessing the inherent sparsity and event-driven nature of SNNs^24,25^, SPEDI employs a selective propagation mechanism, as detailed within Fig. 1. Neural activation depends on reaching a threshold and optimising computational efficiency. This approach considerably reduces the system’s computational overhead and latency, enhancing its overall throughput.

### Neural network comparison

The study compares two distinct computational frameworks: the event-based SCNN (SPEDI) and a traditional non-spiking CNN. Additionally, a version of the SCNN trained through rate-based conversion is also tested which we refer to as Converted SCNN. Unlike typical SCNNs, the conversion variant processes complete data sequences at each computational step, running for a predetermined number of time steps (60 in this study). Hence, this approach processes data in a synchronous manner, in the same way as the CNN. This hybrid approach gains the benefits of spiking computation but retains the drawback of discrete fixed-rate histograms.

On top of that, to verify the ability of the models to map from 1D data to 3D authentically, a Nearest Neighbours baseline is employed. This approach involves selecting the closest histogram from the training set for a given input histogram and assessing its associated ground truth depth image. The effectiveness of this method provides a baseline against which the performance of the neural network models can be compared. Outperforming Nearest Neighbours demonstrates that the approach is not simply memorising training data, but generalising.

The neural network architecture remains constant across all the variations.

**Fig. 3:**
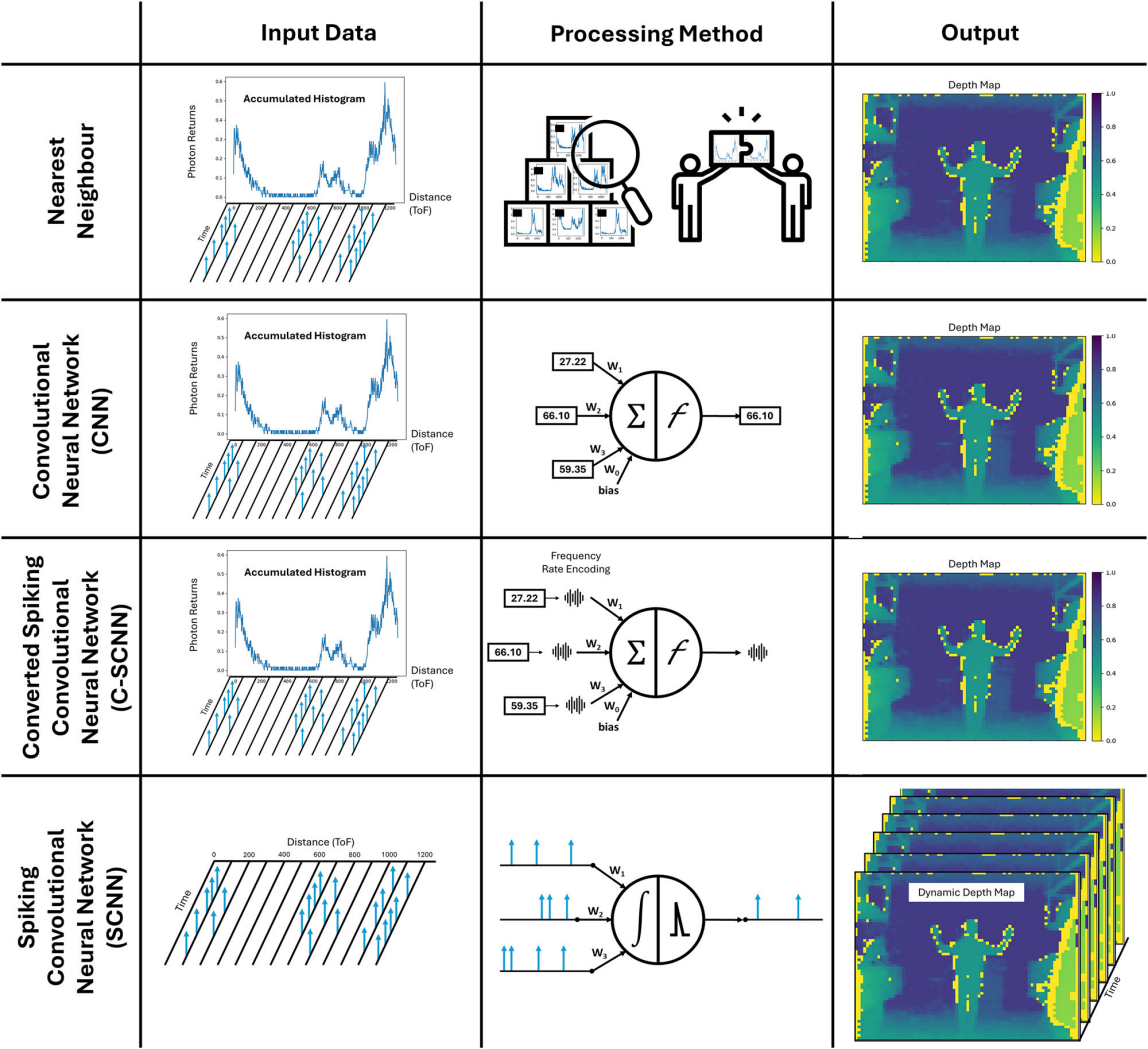
Overview of evaluation methods with respective input data, processing techniques, and output formats. The Nearest Neighbour method uses fixed-rate accumulated histograms as input (photon counts normalized), identifying the closest histogram match from the training set, it then uses the associated depth map for comparison. The CNN approach also takes accumulated histograms, which are then processed through a neural network to compute a discrete depth map image. The Converted-SCNN (C-SCNN) is provided with these histograms as well and involves converting a trained CNN into a rate-based SCNN, resulting in a discrete depth map image. In contrast, SPEDI, our SCNN framework, directly processes event-driven, time-correlated photon returns using spiking neurons that intrinsically accumulate histogram information, thereby generating a dynamic, event-driven depth map that continually updates throughout the SCNN processing.

The evaluations of the networks is carried out by measuring the Mean Squared Error (MSE) and Mean Absolute Error (MAE) between the ground truth depth maps and the images reconstructed from SPI sensor data. An overview of the comparison is detailed in Fig. 3, highlighting the input, output and processing type. Detailed descriptions of the methodologies, including system variants such as the fully neuromorphic SCNN, the traditional CNN, and the rate-based Converted-SCNN (C-SCNN) are elaborated on in the Methods.

### Experimental results

The depth map reconstructions conducted by the SCNN, CNN, rate-based converted SCNN, and Nearest Neighbour methods were subject to both quantitative and qualitative evaluations. These evaluations encompass performance metrics and visual representations to characterise each method’s effectiveness fully.

**Fig. 4:**
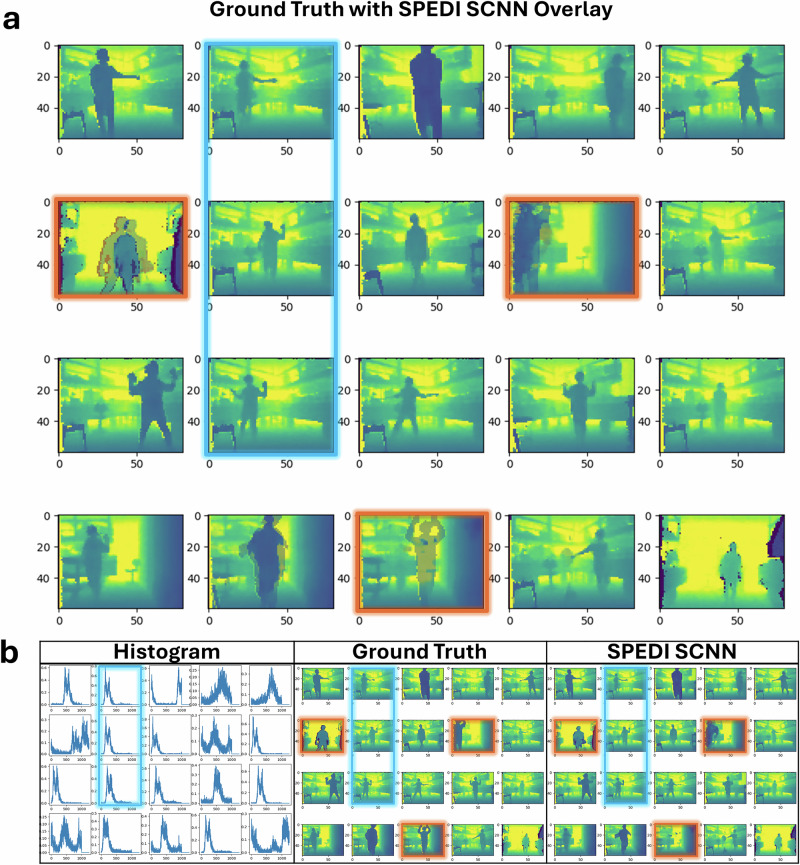
Comparison of results focusing on SPEDI SCNN. **a** Highlights a comparison of the network output compared to ground truth. The network output is overlaid so mistakes appear as partially transparent elements. The side-by-side comparison is shown in (**b**), along with the histogram to get an understanding of the difference in histograms for each scene. Across both (**a**) and (**b**) orange highlights mistakes, while blue highlights how similar-looking histograms can create different images.

Table 1 presents a comparative analysis of the performance metrics. The three networks outperform the Nearest Neighbours baseline, demonstrating generalisation, with the CNN achieving the lowest MSE and MAE values. Figure 4 presents reconstruction examples. Despite the CNN’s numerical advantage, the visual outcomes indicate that the SCNN variants also effectively reconstruct depth maps, accurately capturing both foreground and background details across multiple scenes.

**Table 1:** Comparison table of the different systems for two different metrics

System	MSE (×10^−3^)	MAE (×10^−3^)
CNN	5.3	22
Converted SCNN	9.1	47
SCNN (ours)	9.5	44
Nearest neighbours	24	63

Reconstruction error as Mean Squared Error (MSE). Conversion SCNN stands for the spiking network trained through Nengo conversion. SCNN stands for the spiking network trained through a surrogate gradient in Pytorch. ANN stands for the non-spiking network. All neural networks share the same convolutional architecture. Nearest Neighbours selects the image corresponding to the closest histogram in the training set.

The differences in reconstruction error between SCNN and CNN are justified by their computing principles. For both networks, the returned depth map *y* is the output *y* = *w*_*j*,*i*_*x* of the last convolutional layer of weights *w*_*i*,*j*_, but the SCNN communicates information through spikes, therefore *x* ∈ {0, 1}, while for the CNN, the input will be a continuous floating point number $x\in {\mathbb{R}}$. This means that the granularity of output values in the SCNN will depend fully on the weights of the last layer, while the CNN will receive granularity embedded in the floating point number from the previous layers. The SNN will be able to compensate for this using the time dimension, as its output is the sum of the last layer’s output at each time-step. Still, our simulations only run the SNN for 10 discrete time-steps, therefore, the space of solutions (*w*_*i*,*j*_ configurations) that minimise the loss will be smaller for the SCNN than for the CNN, and therefore harder to find for the optimizer.

These results demonstrate how an SCNN can achieve successful 3D reconstructions in SPI. Unlike traditional CNN approaches, the spike-based network allows to process the input as an asynchronous event stream, outputting results dynamically without the need to wait until measurements are considered complete.

### Impact of single-pixel event-driven imaging

**Fig. 5:**
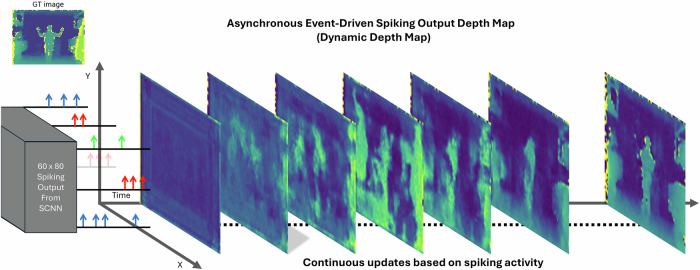
The output of SPEDI. This demonstrates the asynchronous pixel updates in the depth map (60 × 80 resolution) by the SCNN, showing continuous scene updates on a per-photon basis. Initially, the image starts as blank and progressively fills in detail as more photon information is processed through the network. A ground truth image is included for comparison, illustrating the accuracy and dynamic evolution of the depth map as the scene is rendered.

Exploring SPEDI’s SCNN results further, the dynamic advantages of its event-driven approach are highlighted in the real-time updating of depth maps. Figure 5 demonstrates how SPEDI’s SCNN processes incoming data asynchronously and continuously, allowing each pixel update to be triggered by the internal propagation of spikes within the network. As seen in the figure, there is some initial latency to construct the first estimate of the scene; However, this is minimal, and the first reasonable estimate of the scene is constructed within (number of pulses used *12.5 ns). Thus, the effective frame rate can reach 80 MHz but there is the potential for motion blurring effects to occur on timescales of (number of pulses used *12.5 ns). On average ~4000 single photon events were measured corresponding to each ground-truth image collected by the 3D camera. All available events were used to construct the images shown in Figs. 4 and 5, corresponding to an initial estimate of the scene. However, previous work shows that this number of events can be reduced to at least 1000 whilst still being able to retrieve qualitatively reliable results^12^.

These real-time updates are possible because SPEDI’s SCNN naturally accumulates events in its membrane potentials. Therefore it can constantly produce an output, dynamically modifying it with new upcoming events. Moreover, these computations can be performed fully asynchronously in a neuromorphic hardware implementation. On the contrary, non-spiking CNN implementations need to define fixed-rate intervals to perform reconstruction, where they run a full forward pass with the full collection of events. In this work’s setup, the LiDAR acquires data at 80 MHz, while a network forward pass takes on average 20 ms; therefore, performing a forward pass for each new event is completely unfeasible.

Additionally, accumulating frames for processing introduces the challenge of optimising photon accumulation, as too little are not informative, and too many cause motion blur. Asynchronous processing allows the SCNN to propagate information when it reaches neuron thresholds, hence it learns through its weights how much to wait.

The SPEDI algorithm presented in this work, demonstrates the feasibility of a fully event-based approach to SPI. Its implementation in GPU showcases its accuracy and capacity for the generation of dynamic results. In prospective neuromorphic hardware implementations, its latency will be defined by the physical speed of the hardware, and the frequency for the dynamic output update will potentially be as fast as the LiDAR repetition rate, as analog neuromorphic hardware can compute fully asynchronously. As a final summary of the computational benefits, Table 2 compares the computational load and latency of the CNN versus the SCNN. As established in SNN literature, Floating Point Operations (FLOP) are not a suitable metric for neuromorphic hardware. Instead, Synaptic Operations (SyOps) are used^26^, which measure the updates in the neurons of a layer triggered by a spike from the previous layer. As shown in the table, the number of updates in a neuromorphic system can range from zero to the maximum, depending on the sparsity of the input and the network’s activity. In contrast, dense computing will always involve the maximum number of updates for each computation. As explained in the results section, in our simulation, we already proved that the SCNN can compute photon histograms at very high frequency (520 Hz), as opposed to the CNN, hence the note indicating “520 Hz simulation".

**Table 2:** Computational benefits of a neuromorphic implementation compared to GPU computing with an Nvidia A100

Metric	CNN	SCNN
SynOps/pass	5.6M	0–5.6M
Max frequency	52 Hz	80 MHz (520 Hz simulation)

"SynOps/pass” indicates SynOps per forward pass. “Max frequency” indicates the forward pass frequency achieved in GPU by the CNN compared to the potential maximum for the neuromorphic implementation.

SPEDI’s continuous interaction with the dynamic scene environment allows for more timely and accurate depth perception, crucial for applications requiring immediate response to changes, such as in autonomous vehicle navigation or advanced surveillance systems. Future work will aim to optimise this technology further, improving its resolution and scalability to meet broader application needs.

## Discussion

SPEDI represents a considerable advancement in low-latency imaging with single photons. It efficiently produces depth maps from less than complete histogram data, demonstrating substantial data efficiency and an ability to respond swiftly to scene changes. This capability underscores the technology’s potential for settings where rapid data processing is more critical than real-time imaging.

This technology challenges traditional imaging methodologies that rely on static, interval-based data capture, offering a model of continuous interaction with the environment. While not directly enhancing image accuracy, the ability to process with lower latency throughput and less data ensures timely adjustments to dynamic environments.

The low-latency throughput of SPEDI is particularly relevant in applications such as autonomous driving and advanced surveillance, where the ability to quickly adapt to environmental changes can considerably influence operational safety and efficiency. Furthermore, the system’s data efficiency supports extended operation in battery-powered or remote devices, which is crucial for neuromorphic deployments aimed at pushing complex processing and understanding to edge devices.

While SPEDI demonstrates substantial benefits, enhancing its resolution and scalability is essential for broader applications. The availability of neuromorphic devices capable of supporting such advanced systems is limited, posing a challenge to widespread implementation.

However, this initial implementation, while promising, is not yet deployed on neuromorphic computing hardware. Currently simulated on CPU/GPU platforms, the system operates in discrete time steps rather than in true continuous time. Transitioning to neuromorphic hardware would enable truly asynchronous processing and realise the full potential of energy savings through sparse communications inherent in such systems. Neuromorphic computing hardware is a rapidly developing area with the commercialisation of more neuromorphic processing chips on the horizon.

Additionally, SPEDI faces the ‘mirroring problem,’ a fundamental challenge related to the inverse retrieval problem of encoding spatial information only in the time-of-flight^11,23^. Points mirrored across the centre axes of the x and y planes produce identical histograms unless distinct background elements are present to differentiate them. This issue complicates the depth map reconstruction, potentially affecting the accuracy of foreground and background differentiation.

Future research will focus on optimising the architecture of the SCNN to enhance its processing capabilities and adapt more complex network architectures. This involves investigating the integration of advanced materials or newer photonic technologies to further enhance responsiveness and efficiency. Additionally, exploring the application of this technology to other types of sensory data beyond imaging could lead to new avenues in sensor technology.

Ultimately, SPEDI introduces a new paradigm for sensing that utilises photon-event-driven data, setting a foundational technology that promises to expand capabilities in various fields. Its development represents a crucial step toward adaptive and intelligent imaging systems, opening a path to new sensing methodologies that capitalise on the advantages of neuromorphic computing.

## Methods

### Data acquisition

Experimental data was acquired in a scheme similar to that of Turpin et al.^11^. The laser source was a Coherent Chameleon Discovery frequency doubled to 520 nm delivering ~100 fs pulses at a rate of 80 MHz. A 50 degree glass diffuser was used to evenly flood illuminate the scene, with average illumination power of 10 mW (This ensures the system is a class I laser system with a Nominal Ocular Hazard Distance of less than 3 cm from the diffuser). The detector was a SPAD23 single photon detector array from Pi Imaging. No collection optics were used in front of the detector such that all pixels received approximately the same illumination from the scene allowing the array to be used as a single bucket detector. This was done to increase the photon count rate and circumvent the dead time of the individual SPADs. In-built TDCs in the detector were used to retrieve the photon timestamps. An Intel RealSense was used to collect depth maps of the scene for training the SCNN.

The scenes are set in an Indoor environment with low ambient light levels. Field of View is limited by the area covered by the laser which in this specific configuration is around 3.5 m at a distance of 4 m from the system. We note that this can be increased by changing the illumination optics. It is anticipated that the system would be able to adapt well to changes in ambient light as there is no temporal correlation between the ambient light and the laser pulse (it results in a constant background added to the histograms). This, however, has not been tested in practice.

### Neural networks

All tested networks, spiking and non-spiking, follow the same architecture, using a 1D convolution encoder and 2D convolutional decoder, as specified in Table 3.

**Table 3:** Architecture summary

Layer type	Filters	Kernel size	Strides	Padding
Conv1D	128	3	1	Same
Conv1D	256	5	2	Same
Conv1D	256	5	2	Same
Conv1D	512	5	2	Same
Conv1D	512	5	2	Same
Conv1D	512	5	2	Same
Conv1D	512	3	2	0
Conv1D	512	3	1	0
Conv1D	512	3	1	0
Conv1D	512	4	1	0
Reshape	–	–	–	–
Conv2D	512	5	1	Same
UpSampling2D	–	–	–	–
Conv2D	512	5	1	Same
UpSampling2D	–	–	–	–
Conv2D	512	5	1	Same
UpSampling2D	–	5 × 5	–	–
Conv2D	256	5	1	Same
Conv2D	256	3 × 3	1	Same
Conv2D	256	3 × 3	1	Same
Conv2D	128	1 × 1	1	Same
Output Conv2D	1	1 × 1	1	Same
Sigmoid	–	–	–	–

"Same” indicates that padding was added to avoid dropping the rightmost values. Reshape re-arranges the input to dimensions (512, 3, 4).

In the case of the CNN architecture, all convolutions but the last are followed by a Rectified Linear Unit (ReLU) activation function. The final output is bounded to [0, 1] through a sigmoid function. The SCNN on the other hand, uses a spiking neuron where the ReLU function would have been, where the convolution output can be seen as the voltage input to the spiking neuron layer.

Spiking neurons were modelled following the Leaky Integrate-and-Fire (LIF) definition (Eq. (1)). Let *i* be a post-synaptic neuron, *u*_*i*,*t*_ is its membrane potential, *o*_*i*,*t*_ its spiking activation and *λ* the leak factor. The index *j* belongs to the pre-synaptic neuron and the weights *w*_*i*,*j*_ dictate the value of the synapses between neurons (corresponding to the weights of the convolutional layers). Then, the iterative update of the neuron activation is calculated as follows:

1 $${o}_{i,t}=g\left({\sum }_{j}({w}_{ij}{o}_{j,t})+\lambda \cdot {u}_{i,t-1}\right)$$

where *g*(*x*) is the thresholding function, which converts voltage to spikes, given a threshold voltage *U*_*th*_ (we set *U*_*th*_ = 1 in our experiments):

2 $$g(x)=\left\{\begin{array}{cc}{{1}}, & {{{\rm{if}}}}\,x\ge {U}_{th} \hfill \\ {{0}}, & {{{\rm{if}}}}\,x < {U}_{th} \hfill \end{array}\right.$$

After spiking, we reset by subtraction, updating the membrane to ${u}_{i,t}^{* }$ following: ${u}_{i,t}^{* }={u}_{i,t}-{U}_{th}$.

Training was performed in Pytorch for 150 epochs using a batch size of 64, learning rate 1 × 10^−4^, Adam optimizer and a 10% validation split and 20% test split. For the SCNN, a surrogate function was used to make the spiking function differentiable, following Eq. (3) ^27^. We set *α* = 1, which sets the gradient closest to a ReLU function when the threshold is 1.

3 $$\frac{\partial {o}_{t,i}}{\partial {u}_{t,i}}=\alpha \max \{0,1-| {u}_{t,i}| \}$$

## Data Availability

All the data used in this work is available at 10.6084/m9.figshare.30265702.v1.

## Code availability

All code used in this work is available at https://github.com/VicenteAlex/SPEDI.
